# Expression of liver alpha-amylase in obese mouse hepatocytes 

**Published:** 2016

**Authors:** Zohreh Afsartala, Sanaz Savabkar, Ehsan Nazemalhosseini Mojarad, Vahideh Assadollahi, Shima Tanha, Khosro Bijangi, Mohammadreza Gholami

**Affiliations:** 1 *Department of Biology, Science and Research Branch, Islamic Azad University, Tehran, Iran*; 2 *Basic and Molecular Epidemiology of Gastrointestinal Disorders Research Center, Research Institute for Gastroenterology and Liver Diseases, Shahid Beheshti University of Medical Sciences, Tehran, Iran*; 3 *Gastroenterology and Liver Diseases Research Center, Research Institute for Gastroenterology and Liver Diseases, Shahid Beheshti University of Medical Sciences, Tehran, Iran*; 4 *Cellular and Molecular Research Center, Kurdistan University of Medical Sciences, Sanandaj, Iran*; 5 *Department of pharmaceutics, Faculty of Pharmacy, Tehran University of Medical Sciences, Tehran, Iran*; 6 *Tashkhis Baft Aragene Company Ltd (TBA), Tehran, Iran*; 7 *Hepatitis Research Center, Lorestan University of Medical Sciences, Khorramabad, Iran*; 8 *Deptartment of Anatomical Sciences, Lorestan University of Medical Sciences, Khorramabad, Iran *

**Keywords:** Hepatic alpha amylase, Gene expression, Obesity, Flow cytometry

## Abstract

**Aim:**

The aim of this study is to demonstrate the relation between the expression of liver alpha-amylase and obesity.

**Background:**

Alpha-amylase catalyses the hydrolysis of 1, 4-alpha-glucosidic linkages in polysaccharides and has three main subtypes, including: salivary, pancreatic, and hepatic. Hepatic alpha-amylase is involved in glycogen metabolism, and has a role in obesity and its management. In this study, we aimed to analyze the expression of liver alpha-amylase in overweight and obese mouse.

**Material and methods:**

In this study, NMRI male mice were randomly divided into two groups. The sample group (obese) took a high-fat and carbohydrate diet, while the control group (normal) took a laboratory pellet chow for eight weeks. During this period, their weight was measured. After eight weeks, liver hepatocytes were isolated using an enzymatic digestion method. Immunocytochemistry (ICC) and flow cytometry analysis were performed to measure alpha amylase protein expression in mouse liver hepatocyte cells.

**Results:**

A significant difference in the body weight was observed between the two groups (p<0.05). The qualitative protein expression of liver alpha-amylase was found to be higher in the obese group in both tests (immunocytochemistry and flow cytometry). Animals from the test group presented higher alpha-amylase expression, which suggests that this hepatic protein may constitute a potential indicator of susceptibility for fat tissue accumulation and obesity. The present data demonstrates an increased expression of liver amylase in obese mice.

**Conclusion:**

These results suggest that liver amylase secretion might be useful for predicting susceptibility to obesity induced by consumption of a high-fat and carbohydrate diet.

## Introduction

 Alpha-amylases (1, 4-aD-glucan-4-glucanohydrolase, EC 3.2.1.1) are widely found in microbial species, plants, and animals. They are involved in the hydrolysis of amylum and glycogen (1, 2). Three amylase isoforms were found in humans and other animals. These isoforms are secreted mainly by the salivary glands and the pancreas, and partly by the liver. Alpha amylase plays an important role in the digestion of amylum polysaccharides and is the main source of glucose production in blood (3)

The prevalence of obesity has been increased enormously worldwide due to an imbalance between energy intake and output, and consumption of high amounts of fat. A high-fat diet increased plasmatic levels of triacylglycerol and total cholesterol, as well as the risk of insulin resistance and cardiovascular disorder (4).

Salivary amylase (SA) hydrolyses the alpha-1 and 4 glycosidic linkage of starch in the mouth and converts it to oligosaccharides (5). The rest will be digested in the intestine by pancreatic amylase (PA) and hepatic alpha-amylase (HA) hydrolysis activity to maltose, maltotriose, and other oligomers that are eventually converted to the glucose (6, 7).

The alpha-amylase genes cluster is a suitable experimental tool for studying the tissue specificity of gene expression. Members of this cluster are expressed in different tissues, such as the parotid gland, the pancreas, and the liver. Two different types of alpha-amylase genes are expressed in the mice of the inbred strain. The single-copy gene Amy-l expresses two types of mRNA with identical coding, but different 5´-terminal non-coding sequences in the parotid gland and the liver (8). The tissue-specific leader sequences are specified by two separate Amy-1 exons, which contain the cap sites for the two different mRNAs (9).

The parotid gland-specific mRNA has a concentration that is at least 100-fold higher in the parotid gland than in the liver tissue (10). The different concentrations of Amy-l-related mRNAs in the parotid gland and the liver are regulated primarily at the transcriptional level by two promoters with different activities (11). The less active promoter directs the synthesis of liver-type mRNA and is active in both tissues. An approximately 30 times more active promoter exists in the parotid gland. This promoter directs the synthesis of mRNA with the parotid-type leader sequence. Amy-2 specifies that pancreatic alpha-amylase mRNA exists as a multiple. Amy-2 has similar copies in the mouse genome (12). The pancreas-specific mRNA is about 10 and 1,000 times more abundant than its parotid and liver counterparts, respectively (10).

In obesity, a high glycogen content is hydrolysed by the activity of pancreatic and salivary alpha-amylase, which leads to an increased level of glucose in the blood (13). The studies showed that hepatic amylase might also have a role in the metabolism of glycogen as it has a high affinity to this carbohydrate. The level of blood glucose increases in metabolic diseases such as obesity and type 2 diabetes (14). Therefore, controlling amylase activity could be an important approach for diagnosis and treatment of high blood glucose (15, 16). In this regard, amylase inhibitors prevent the hydrolysis of starch into oligomers (17). These inhibitors could ameliorate the deleterious effects of low blood sugar after meals to delay the glucose absorption in the blood (18). Inhibition of alpha-amylase significantly decreases the postprandial increase of blood glucose after a mixed carbohydrate diet. Thus, it can be used as a method to control postprandial blood glucose levels in obese and type 2 diabetic patients (19).

Obesity has a significant incidence in the world, which has been led to clinical complications with an economic cost to patients. Due to limited studies in this research area, in this study, we aimed to discuss the expression level of liver alpha-amylase in obese mouse hepatocytes. In this study, we compared the expression level of alpha-amylase in obese mice and the control group to evaluate the role of liver alpha-amylase in obesity. The results of this study will be important for treating obesity and reducing its complications. 

## Material and Methods

**Animal Model**

NMRI male mice (six weeks old) were randomly divided into two groups (n=6). The sample group (obese) took a high-fat and carbohydrate diet for eight weeks. Meanwhile, the control group (normal) took laboratory pellet chow. Over an eight-week period, the rats’ body weight was measured weekly. NMRI mice (25–30 g) were kept in propylene cages at room temperature for 12 h light/dark cycles with chow and water. Experimental protocols were approved by the ethical committee of Azad University on Care and Use of Animals. This was in accordance with the guidelines of the National Institute of Health, USA (20). 

**Diet Composition**

The commercial diets used for animal studies consisted of 19% protein, 56% carbohydrate, 3.5% lipids, 4.5% cellulose, and 5% vitamins and minerals, with a total energy content of 17.03 kJ/gr. The standard high-fat and carbohydrate diet used for the study comprised the following hyper-caloric constituents: 15 g of laboratory animal chow, 10 g of roasted groundnuts, 10 g of milk chocolate, and 5 g of maize biscuits. These ingredients were mixed and prepared in the form of pellets containing 20% protein, 48% carbohydrate, 20% lipids, 4% cellulose, as well as 5% vitamins and minerals. The total energy content of this diet was 21.40 KJ/g. Thus, compared to the normal diet, the high-fat and carbohydrate diet was hyper-caloric with a net energy difference of 4.37 kJ/g. To avoid lipid peroxidation, the food stock was stored at 24◦C (20).

**Isolation and Culture of Mouse Hepatocytes**

Liver tissue was washed with cold PBS containing penicillin-streptomycin 2X in sterile conditions, thus removing blood content. Further, cool PBS with antibiotics was drawn into a 5 ml syringe, and then the tip of the needle was inserted into the hepatic vein to remove more blood. Then, it was transferred into a DMEM medium. The tissue was then cut into smaller parts using sterile scissors and a surgical blade (21).

Next, the cells were harvested using an enzymatic method. In short, 5 ml (Trypsin-EDTA) was added to the samples followed by 5 min of incubation at 37°C. To neutralize the proteolytic activity of trypsin, a culture medium containing 10% FBS was added and cells were centrifuged for 5 min at 1,500 RPM. After 5 min, the supernatant was discarded and a fresh medium containing 10% FBS was added. The cells were then transferred to the culture flask. A small amount of cell suspension was removed for the cell count and investigating the percentage of cell viability using the dye exclusion method (21).

**Immunofluorescence Microscopy**

Cultured cells on coated glass coverslips were fixated with cold absolute ethanol for 10 min. Given that the selected markers were cytoplasmic proteins, Triton X-100 was used to permeablize the cells for 15 min. To block the non-specific binding, 5% goat serum was used, followed by 45 min of incubation at room temperature. After washing with PBS thrice, the cells were incubated with the rabbit anti- mouse alpha-amylase antibody (1/100, Santa Cruz biotech) for 3 h. The cells were then incubated with FITC-conjugated anti-rabbit IgG (1/100, Santa Cruz biotech) for 1 h. The cells were protected from drying by adding 1 ml PBS to each well and keeping this at 4°C until it was observed through an inverted fluorescence microscope. The samples were examined using a fluorescence microscope (Nikon, Melville, NY) (22).

**Flow Cytometry Assay**

Flow cytometry assay was done as follow: Suspend cell pellet in 5 ml of PBS and centrifuge for 5 min at 1,500 RPM and fix with araformaldehyde 4% (4ºC), followed by 10 min of incubation with Triton X-100.

The cells washed twice with goat serum and incubated with a rabbit anti- mouse alpha-amylase antibody overnight (1:50 Santa Cruz biotech). After washing thrice, the samples were incubated for 45 min with FITC-conjugated anti-rabbit IgG (1/100, Santa Cruz biotech). Cell suspension analyses were performed using BD flow cytometry (23).

**Statistical Analysis**

The results are presented as means ± standard deviation. The data was analysed by a student’s t-test or a one-way analysis of variance (ANOVA), followed by Tukey’s test, using the GraphPad Prism program (version 4.0). Differences were considered significant at p<0.05. 

## Results

**Figure 1:**
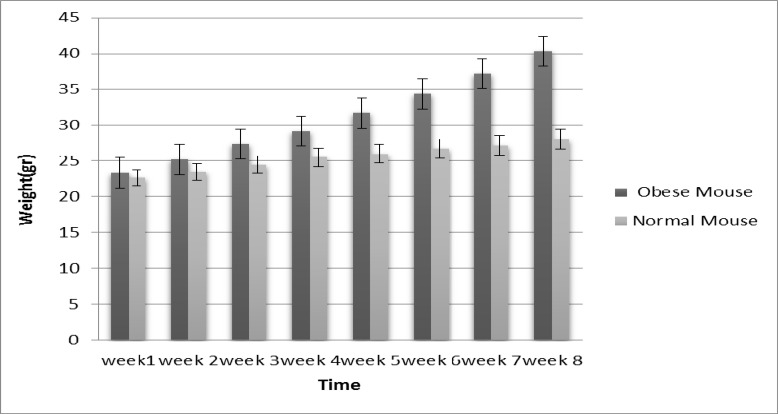
The diagram of weight Change (obese & control groups) of mice under different diets composition; A significant increase in weight mean change were observed in obese group (6mice) compared to the control group (6 mice

**Figure 2:**
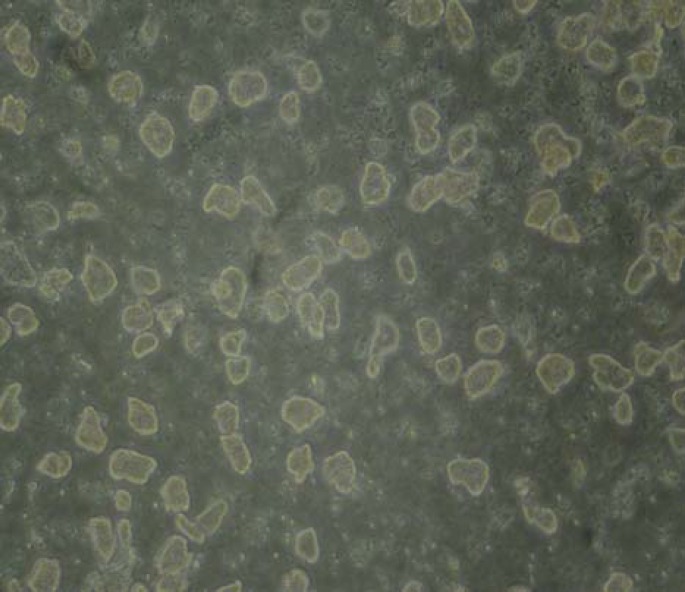
Mouse hepatocyte primary cells. Magnification; 10X (NIKON ECLIPSE E600

Abdominal obesity was obtained in NMRI mice after eight weeks of a high-fat and carbohydrate diet. The body weights of the mice and visceral obesity (abdominal) confirmed the incidence of obesity in mice taking a high-fat and carbohydrate diet. The results showed a significant difference between the weight of mice with a high-fat and carbohydrate diet and control group mice with a normal diet (p<0.05) (Figure 1). There are limited studies on the isolation of the hepatocyte primary cell culture. The mouse hepatocyte primary cell was cultured as described previously. The result is shown in Figure 2. The viability percentage of the cells was assessed using the trypan blue dye exclusion test. If the viability was less than 85%, the cell was not utilized in the test.

**Figure 3:**
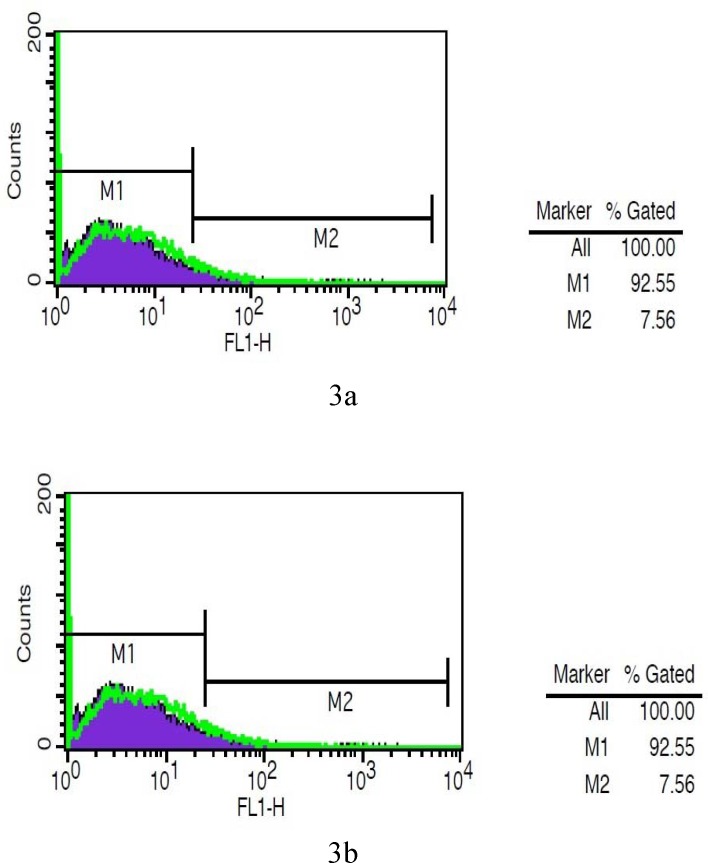
Flowcytometry analysis showing expression of Alpha amylase in hepatocyte of obese (A) and normal mouse (B); the purple graft represented the isotype control.

**Figure 4:**
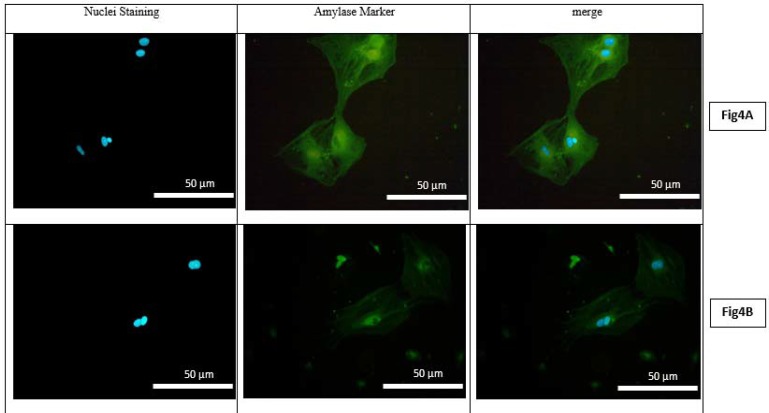
Immunoflurecent assay representing the low expression of amylase in the hepatocyte of normal mouse (Fig 4A) and the high expression of amylase in the obese mouse (Fig 4B) amylase expression is obvious in FITC stained cell (green) and nuclei are stained with DAPI (blue). Observation under the immunofluorescent microscope Magnification; 20X(NIKON ECLIPSE E600

Flow cytometry analysis of amylase indicated that the expression level of this gene in hepatocytes derived from mice receiving a high-fat and carbohydrate diet was 48% (Figure 3A). However, the expression level of this gene in hepatocytes derived from control mice (normal diet) was about 7% (Figure 3B). The results showed a direct correlation between alpha-amylase gene expression in the liver and the increase of a high-fat and carbohydrate diet and the resultant obesity. The data obtained by flow cytometry was confirmed by immunocytochemistry (ICC) staining. ICC results revealed that amylase gene expression in hepatocytes derived from the liver of mice receiving a high-fat and carbohydrate diet was higher than that of control mice receiving a normal diet (Fig 4A, 4B). 

## Discussion

The existence of individuals with a tendency and individuals with a resistance to obesity has been noted for both humans and animals. In rats, this differential response to weight changes has already been seen in individuals on high-fat diets, and has been attributed to different causes, such as overconsumption, or to the modulation of gene expression related to lipogenesis (24-26).

The liver produces a variety of proteins with important roles in the function of the body, some of which have been suggested to influence digestive behavior. 

The interest in the study of liver proteins has increased. These proteins are a source of biomarkers of pathological disorder and may help in the understanding the molecular mechanisms of the disease.

 Nonetheless, no studies were found on the hepatic amylase function in the obesity.

The expression of alpha-amylase in the liver and serum was studied for the first time in 1961. This study showed that the enzyme in the liver has an antigenic properties similar to the enzyme expressed by saliva. Moreover, many studies have stated that the existing amount of amylase in serum is supplied by the liver rather than other sources such as saliva or the pancreas in normal physiological conditions (27).

McGeachin and Potter (1961) reported that chemical damages to the liver tissue leads to a considerable reduction of amylase activity in the liver and serum in vivo (28). It has also been reported that liver amylase in the blood of rats receiving a nutritious diet is comprised of serum amylase and glycogen (29). Although the amylase expression by the liver has been proven and the nucleotide sequence of this enzyme in mice has been determined, no absolute performance has yet been identified for this enzyme (8). 

However, some studies have noted that this enzyme plays a major role in glycogen metabolism in the liver (30).

The role of hormones in liver alpha-amylase activity is well known. It has been found that adrenaline injections increase the activity of alpha-amylase in serum. Meanwhile, glucagon has the opposite effect, as it reduces the enzyme activity of alpha-amylase (31). Studies showed that in damaged hepatocytes of patients, glucagon would not reduce the alpha-amylase activity. This suggests a role for liver alpha-amylase in the glycogen metabolism (32).

In 2001, while studying the alpha-amylase gene expression in rat liver, Koyama et al. stated that this enzyme is glycosylated with the molecular mass of 50 kDa and has a strong affinity to glycogen (33). There is a result indicating that the liver is the major source of serum amylase rather than the pancreas or the parotid glands (27).

The serum amylase level is influenced by many factors, such as hydration status, psychosocial stress, and dietary habits. It has been shown that the average copy number of the AMY1 gene is higher in populations that evolved under high-starch diets versus low-starch diets, thus reflecting an intense positive selection imposed by diet on amylase copy number during the evolution. As described earlier, AMY1 is a gene that specified for coding of salivary and hepatic amylase (34).

According to the results of the present study, it was demonstrated that the average expression level of the liver alpha-amylase gene in the hepatocytes of mice receiving a high-fat and carbohydrate diet was higher than that of mice receiving a normal diet. This increase in the protein expression was also confirmed by flow the cytometry and immunocytochemistry. Our result showed the high expression of liver alpha-amylase in the hepatocytes of obese mice. The finding indicates the role of this enzyme in glycogen metabolism.

Up-regulated amylase in serum of mice receiving a high-fat diet compared with the control group might indicate that the liver iso-enzyme has a direct correlation with obesity and glycogen increase.

In conclusion, consumption of a high-fat diet induced obesity in NMRI mice. It is concluded that liver alpha-amylase (or serum amylase) may be useful to predict susceptibility of obesity. Liver alpha-amylase levels in animals are related to the rate of future weight gain. However, the mechanisms responsible for the expression of liver alpha-amylase in line with obesity susceptibility need to be investigated. This study suggested the liver alpha-amylase gene as a molecular biomarker of obesity. Therefore, liver alpha-amylase could be considered as an indicator for the identification of obesity and other metabolic diseases.

## References

[1] Muralikrishna G, Nirmala M (2005). Cereal α-amylases—an overview. Carbohydr Polym.

[2] Qin X, Ren L, Yang X, Bai F, Wang L, Geng P (2011). Structures of human pancreatic α-amylase in complex with acarviostatins: Implications for drug design against type II diabetes. J Struct Biol.

[3] Butterworth PJ, Warren FJ, Ellis PR (2011). Human α‐amylase and starch digestion: An interesting marriage. Starch‐Stärke.

[4] Berghöfer A, Pischon T, Reinhold T, Apovian CM, Sharma AM, Willich SN (2008). Obesity prevalence from a European perspective: a systematic review. BMC Pub Health.

[5] Lehmann U, Robin F (2007). Slowly digestible starch–its structure and health implications: a review. Trends Food Sci Technol.

[6] Li C, Begum A, Numao S, Park KH, Withers SG, Brayer GD (2005). Acarbose rearrangement mechanism implied by the kinetic and structural analysis of human pancreatic α-amylase in complex with analogues and their elongated counterparts. Biochemistry.

[7] Kajaria D, Ranjana JT, Tripathi YB, Tiwari S (2013). In-vitro α amylase and glycosidase inhibitory effect of ethanolic extract of antiasthmatic drug—Shirishadi. J Adv Pharm Technol Res.

[8] Hagenbüchle O, Tosi M, Schibler U, Bovey R, Wellauer PK, Young RA (1981). Mouse liver and salivary gland α-amylase mRNAs differ only in 5′ non-translated sequences. Nature.

[9] Young RA, Hagenbüchle O, Schibler U (1981). A single mouse α-amylase gene specifies two different tissue-specific mRNAs. Cell.

[10] Schibler U, Tosi M, Pittet AC, Fabiani L, Wellauer PK (1980). Tissue-specific expression of mouse α-amylase genes. J Mol Biol.

[11] Schibler U, Hagenbüchle O, Wellauer P, Pittet A (1983). Two promoters of different strengths control the transcription of the mouse alpha-amylase gene Amy-1a in the parotid gland and the liver. Cell.

[12] Schibler U, Pittet AC, Young RA, Hagenbüchle O, Tosi M, Gellman S (1982). The mouse α-amylase multigene family sequence organization of members expressed in the pancreas, salivary gland and liver. J Mol Biol.

[13] Hariri N, Thibault L (2010). High-fat diet-induced obesity in animal models. Nutr Res Rev.

[14] Stene LC, Oikarinen S, Hyöty H, Barriga KJ, Norris JM, Klingensmith G (2010). Enterovirus infection and progression from islet autoimmunity to type 1 diabetes: The Diabetes and Autoimmunity Study in the Young (DAISY). Diabetes.

[15] McCue P, Kwon YI, Shetty K (2005). Anti-diabetic and anti-hypertensive potential of sprouted and solid-state bioprocessed soybean. Asia Pac J Clin Nutr.

[16] Gupta R, Gigras P, Mohapatra H, Goswami VK, Chauhan B (2003). Microbial α-amylases: a biotechnological perspective. Proc Biochem.

[17] Preuss HG, Echard B, Bagchi D, Stohs S (2007). Inhibition by natural dietary substances of gastrointestinal absorption of starch and sucrose in rats and pigs: 1. Acute studies. Int J Med Sci.

[18] Thilagam E, Parimaladevi B, Kumarappan C, Mandal SC (2013). α-Glucosidase and α-amylase inhibitory activity of senna surattensis. J Acupunct Meridian Stud.

[19] Ali H, Houghton P, Soumyanath A (2006). α-Amylase inhibitory activity of some Malaysian plants used to treat diabetes; with particular reference to Phyllanthus amarus. J Ethnopharmacol.

[20] de Melo CL, Queiroz MGR, Fonseca SG, Bizerra AM, Lemos TL, Melo TS (2010). Oleanolic acid, a natural triterpenoid improves blood glucose tolerance in normal mice and ameliorates visceral obesity in mice fed a high-fat diet. Chem Biol Interact.

[21] Li WC, Ralphs KL, Tosh D (2010). Isolation and culture of adult mouse hepatocytes. Methods Mol Biol.

[22] Kojima T, Fort A, Tao M, Yamamoto M, Spray DC (2001). Gap junction expression and cell proliferation in differentiating cultures of Cx43 KO mouse hepatocytes. Am J Physiol Gastrointest Liver Physiol.

[23] Shi J, Aisaki K, Ikawa Y, Wake K (1998). Evidence of hepatocyte apoptosis in rat liver after the administration of carbon tetrachloride. Am J Pathol.

[24] Li H, Xie Z, Lin J, Song H, Wang Q, Wang K (2008). Transcriptomic and metabonomic profiling of obesity-prone and obesity-resistant rats under high fat diet. J Proteome Res.

[25] do Nascimento AP, Monte-Alto-Costa A (2011). Both obesity-prone and obesity-resistant rats present delayed cutaneous wound healing. Br J Nutr.

[26] Clarke S, Turini M, Jump D (1997). Polyunsaturated fatty acids regulate lipogenic and peroxisomal gene expression by independent mechanisms. Prostaglandins Leukot Essent Fatty Acids.

[27] Arnold M, Rutter WJ (1963). Liver amylase III Synthesis by the perfused liver and secretion into the perfusion medium. J Biol Chem.

[28] McGeachin RL, Potter BA (1961). Electrophoretic Behaviour of Rat Serum Amylase. Nature.

[29] Takeuchi T, Matsushima T, Sugimura T (1975). Electrophoretic and immunological properties of liver α-amylase of well-fed and fasted rats. Biochim Biophys Acta.

[30] Rutter WJ, Arnold M, Brosemer RW, Miller J (1961). Liver amylase II Physiological role. J Biol Chem.

[31] Dreiling DA, Janowitz HD, Marshall D, Haemmerli P (1958). Relationship between blood amylase and factors affecting carbohydrate metabolism. Am J Dig Dis.

[32] Dreiling DA, Rosenthal WS, Kass M, Janowitz HD (1959). Relationship between blood amylase and factors affecting carbohydrate metabolism. Am J Dig Dis.

[33] Koyama I, Komine Si, Hokari S, Yakushijin M, Matsunaga T, Komoda T (2001). Expression of α‐amylase gene in rat liver: Liver‐specific amylase has a high affinity to glycogen. Electrophoresis.

[34] Santos J, Saus E, Smalley S, Cataldo L, Alberti G, Parada J (2012). Copy number polymorphism of the salivary amylase gene: implications in human nutrition research. J Nutrigenet Nutrigenomics.

